# Highly efficient and aberration-free off-plane grating spectrometer and monochromator for EUV—soft X-ray applications

**DOI:** 10.1038/s41377-023-01342-9

**Published:** 2024-01-08

**Authors:** Jie Li, Kui Li, Xiaoshi Zhang, Dimitar Popmintchev, Hao Xu, Yutong Wang, Ruixuan Li, Guangyin Zhang, Jiyue Tang, Jin Niu, Yongjun Ma, Runyu Meng, Changjun Ke, Jisi Qiu, Yunfeng Ma, Tenio Popmintchev, Zhongwei Fan

**Affiliations:** 1 Aerospace Information Research Institute, Chinese Academy of Sciences, Beijing, 100094 China; 2 School of Optoelectronics, University of the Chinese Academy of Sciences, Beijing, 100049 China; 3 Yunnan University, Kunming, Yunnan 650500 China; 4 Photonics Institute, TU Wien, Vienna, A-1040 Austria; 5 Yunnan Observatories, Chinese Academy of Sciences, Kunming, Yunnan 650011 China; 6 University of California, Physics Department, San Diego, La Jolla, CA 92093 USA

**Keywords:** High-harmonic generation, X-rays, Spectrophotometry

## Abstract

We demonstrate a novel flat-field, dual-optic imaging EUV—soft X-ray spectrometer and monochromator that attains an unprecedented throughput efficiency exceeding 60% by design, along with a superb spectral resolution of *λ*/Δ*λ* > 200 accomplished without employing variable line spacing gratings. Exploiting the benefits of the conical diffraction geometry, the optical system is globally optimized in multidimensional parameter space to guarantee optimal imaging performance over a broad spectral range while maintaining circular and elliptical polarization states at the first, second, and third diffraction orders. Moreover, our analysis indicates minimal temporal dispersion, with pulse broadening confined within 80 fs tail-to-tail and an FWHM value of 29 fs, which enables ultrafast spectroscopic and pump-probe studies with femtosecond accuracy. Furthermore, the spectrometer can be effortlessly transformed into a monochromator spanning the EUV—soft X-ray spectral region using a single grating with an aberration-free spatial profile. Such capability allows coherent diffractive imaging applications to be conducted with highly monochromatic light in a broad spectral range and extended to the soft X-ray region with minimal photon loss, thus facilitating state-of-the-art imaging of intricate nano- and bio-systems, with a significantly enhanced spatiotemporal resolution, down to the nanometer–femtosecond level.

## Introduction

Ever since the first observation of high-order harmonics generation (HHG)^1–4^ using ultrashort ultraviolet (UV) and infrared (IR) laser pulses, this extreme nonlinear upconversion phenomenon has become a practical laboratory-scale technology for producing coherent light in the extreme ultraviolet (EUV) and soft X-ray (SXR) spectral ranges exceeding keV photon energies. In more than three decades of intense research, this light source has evolved into a versatile laser light tool extending across the EUV and soft X-ray region^5–9^ with temporal bursts as short as ~50 attoseconds^10,11^. Such light sources have the capacity to penetrate optically non-transparent objects, allowing for the visualization of subwavelength nanostructures, manipulation of magnetic states and currents, and enabling the study of materials chemistry and structure at scales as precise as nanometers, and temporal resolutions ranging from femtoseconds to attoseconds^12–16^. In addition, the HHG sources can also be designed to have a variable spin and orbital angular momentum states surpassing the tunability of the VIS-IR lasers^17,18^. The advent of the kHz–MHz femtosecond laser amplifiers scaled the average high harmonic power to more than ten milliwatts^19^, thus already advancing a wide range of spectroscopic and imaging applications in the fields of physics, chemistry, and nanoscience. Furthermore, the superior spatial and temporal coherence of the harmonics, compared to other large- and small-scale sources, and their attosecond-to-femtosecond temporal and nanometer spatial resolution has enabled dynamic studies at the space-time resolution extreme of atomic, molecular systems, advanced quantum materials, and nano-devices^12,14,20,21^.

In static and dynamic coherent EUV—X-ray measurements, the transmitted or reflected light is analyzed using two general approaches: an ultrabroad bandwidth EUV—X-ray illumination^22^ or a monochromatic illumination. However, due to the short wavelength of the light (1–100 nm) and the strong material absorption in the EUV–SXR electromagnetic region, the optical elements used in a spectrometer or monochromator such as gratings, steering multilayer mirrors, focusing toroidal or elliptical mirrors typically have low throughput efficiency and high cost. Additionally, the low conversion efficiency of the HHG sources - on the order of a few microwatts or even nanowatts—further complicates experiments where the signal-to-noise ratio is of the essence. Consequently, low photon emission has been the major roadblock in HHG applications. So, while developing a brighter light source is of paramount importance, designing a highly efficient EUV—soft X-ray spectrometer and monochromator is another key to resolving such problems.

Various types of spectrometer and monochromator instruments have been developed for compact- and large-scale sources (Table 1), generally consisting of focusing optics and diffraction gratings that are mainly used at grazing incidence angles to improve throughput performance. The most common configuration of EUV—soft X-ray spectrometer utilizes a single optic, typically either an elliptical, toroidal, or spherical grating with variable line spacing (VLS). Such spectrometers can have either or both imaging and flat-field capability^23,24^. Dual-optic spectrometers and monochromators comprising focusing optics and variable line spacing have been employed to increase flexibility, at the cost of throughput efficiency. A notable example is the Hettrick-Underwood design^25^, a configuration that employs variable line spacing grating and elegantly works within classical geometries’ limitations to achieve enhanced performance while offering flat-field and image-mode capability^26,27^. These designs are primarily employed in synchrotron beamlines and have been successfully adapted for the monochromatization of free-electron laser light. Nonetheless, it’s worth noting that the diffraction efficiency generally falls short when compared to configurations utilizing off-plane geometry^28^.

**Table 1:** Comparison of advanced EUV—X-ray spectrometer and monochromators

Designed function	Spectrometer	Spectrometer/monochromator	Spectrometer/monochromator	Spectrometer/monochromator	Spectrometer/monochromator (this work)
Schematic diagram					
Number of optics	1	1–2	4–6	3	2
Grating type	Variable line spacing grating^23,24^	Variable line spacing toroidal grating^44–46^	Dual plane grating (Conical diffraction)^38–42^	Plane grating (Conical diffraction)^29,31^	Plane grating (Conical diffraction)
Throughput efficiency	5–15%	16% (1 grating) 2.6% (2 grating)	10—18%	~25%	Exp. average 7 HH >40% (Theoretically >60%)
Imaging mode	No	Yes	Yes	Yes	Yes
Spectral resolution (λ/Δλ)	200–1000	~400	~100	>200	>200
Spectral range	1–100 nm (3 gratings)	9–170 nm (3 gratings)	20–400 nm	10–50 nm	10–50 nm
Preserves polarization	N.A.	N.A.	N.A.	Yes	Yes
Temporal dispersion	~1 ps	<11 fs	<5 fs	~100 fs	Tail-to-tail 80 fs FWHM 29 fs

To circumvent such problems, multi-optical element (typically three or more optical elements) spectrometers in off-plane geometry have been explored in various attosecond high harmonic applications^29,30^. The focusing and diffraction functions are separated inside such spectrometers, allowing for a higher diffraction efficiency, better spatial profile, improved spectral resolution, and lower pulse dispersion simultaneously. However, more optical elements typically result in alterations of light phase, polarization, or angular momentum states, leading to reduced throughput efficiency, increased complexity, and the necessity for more intricate alignment procedures. Simplifying the design in off-plane geometry requires innovative methods to overcome their inherent limitations regarding efficiency or resolution.

In this work, we report a simple dual-optic imaging spectrometer-monochromator using a uniformly spaced flat grating in off-plane geometry with globally optimized spatio-temporal parameters for EUV and soft X-ray sources that address most experimental challenges. Our approach simultaneously combines a record-breaking high throughput efficiency, surpassing 40% experimentally or 60% theoretically, a high spectral resolution of $\lambda /\Delta \lambda$ > 200, minimal sub-80 fs tail-to-tail temporal dispersion, with an FWHM duration of under 29 fs, and extensive spectral range coverage—all achieved using just one regular grating while preserving the polarization state within a straightforward geometry. Additionally, this dual-optic system is highly efficient and cost-effective and can serve as a complementary or alternative replacement to the EUV—soft X-ray multilayer mirrors for a wide range of wavelengths. This is particularly valuable because the efficiency of currently available mirrors is, at best, modest, except for a select few wavelengths.

## Results

### Systematic elimination of aberrations in the spatiotemporal domain

**Fig. 1:**
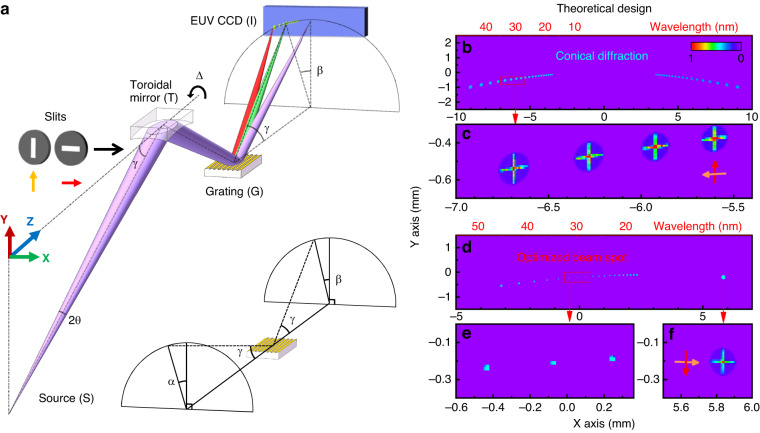
Aberration-corrected spectrometer and monochromator in conical diffraction geometry. **a** The instrument consists of a toroidal mirror, free to rotate around the Z axis, and a plane conical grating. The altitude angle γ, and the azimuthal angles α and β, describe the direction of the incoming and diffracted rays. Horizontal and vertical slits are used in simulation models: the yellow and red arrows show the direction of the vector slits, representing the signs of the aberrations due to the optical elements along the tangential and sagittal directions. **b** Simulated first-order diffraction beam spots of 17–45 nm detected by EUV CCD for toroidal angle $\Delta =0^\circ$. **c** Magnified view of the first-order (*m* = 1) diffracted beam spots with wavelengths close to 30 nm from (**b**). The bright line shows the beam distortion from the tangential and sagittal ray fans. **d** Aberration-corrected first-order beam spots achieved by increasing the toroidal angle Δ from $0^\circ$ to $2^\circ$. The spot near *X* = 6 mm is the zeroth-order beam. **e** Magnified view of the first-order diffracted beam spots with a perfect round shape from (**d**) at ~ 30 nm. **f** Magnified view of zeroth-order beam spot from (**d**)

Blazed gratings can achieve exceptionally high diffraction efficiency of over 90% in the first and higher orders when used in a conical diffraction geometry (see Fig. 1a) since the shadowing effect at low grazing angles is minimal compared to classical diffraction^28^. However, diffraction gratings also introduce aberrations to non-collimated beams. In past approaches, a collimated beam was used to reduce the distortions caused by the grating in a conical diffraction geometry. This requires implementing at least two focusing optical elements in the spectrometer: one set for collimating and another for focusing^29,31^. As a result, the throughput of the optical system is lowered, and the design complexity and alignment are more intricate. Contrary, we demonstrate here, for the first time, that we can correct the aberrations in a dual-optical element system due to the diffraction grating element in a conical geometry by adjusting a single degree of freedom - namely rotating the azimuthal angle of a toroidal focusing element. The optical aberrations are deviations of the wavefront of the light waves from a perfectly spherical shape that are usually expressed as a series of polynomial terms with increasing powers of the radial coordinate, i.e., spherical aberrations, coma, astigmatism, defocusing, etc. However, we substantially minimize the spatio-temporal wavefront error in our dual-optic geometry without targeting any particular aberration term.

In this work, we demonstrate a globally optimized and aberration-free system using a single blazed grating in conical diffraction geometry in combination with a toroidal mirror at a grazing angle (Fig. 1a). We note that the focusing optic can be any other type of curved surface or sets of optical elements forming a 2D focal spot with relatively low aberrations, i.e., elliptical mirror and KB mirror sets. A toroidal mirror has minimal aberrations when used in a 2$f$–2$f$ imaging configuration. However, when such a spectrometer is used in other than 1:1 imaging, a low aberration mirror should be preferred to minimize aberration distortions, i.e., elliptical mirror and diabolic. We start by systematically removing the present aberrations using both analytical and raytracing simulations. For that purpose, we first consider the high harmonic light as a point source with a full divergence angle of $2\theta$. A toroidal mirror with an effective focal length $f$ images the HHG source onto the EUV CCD camera, where the source and the camera are 4$f$ apart. A grating with its grooves parallel to the Z axis is placed halfway between the toroid and the detector. The direction of the incoming and diffracted rays on the grating can be described by two parameters: the altitude incidence angle $\gamma$, which is the angle between the ray and the groove direction, and the azimuthal angle $\alpha$ (or $\beta$), which is the angle between the grating normal and the projection of the incoming and diffracted ray in the plane perpendicular to the grooves.

We simulate the aberrations from the grating alone by tracing the first-order and higher-order beams from the grating. The toroidal mirror is set at the symmetric default configuration with minimal aberration, i.e., $\Delta$ = 0°. Specifically, Fig. 1b shows the simulated wavefront distortions for zeroth and the first diffraction orders of the HHG beam from 17 to 45 nm, as captured on the CCD detector. The grating diffraction angles follow the equation:

1 $$\frac{m\lambda }{d}=\,\sin \gamma (\sin \alpha +\,\sin \beta )$$

where $d$ is the groove spacing of the grating, $\gamma$ is the altitude angle, $\alpha$ and $\beta$ are the azimuthal angles, and $m$ is the diffraction order. Additionally, as a rule of thumb, the maximum efficiency of such grating is roughly at a wavelength of ${\lambda }_{\max }=2d\sin \psi \sin \gamma /m$. The blaze angle $\psi$ satisfies the condition $2\sin \psi =\sin \alpha +\sin \beta$.

We use “vector” slits along the tangential and sagittal directions in front of the optics in our raytracing model to analyze the distortions of the grating and toroidal mirror. The arrow direction and length of the “vector” slits indicate the sign and magnitude of the aberrations, respectively. We define “up” or “right” pointing arrows as positive signs of tangential and sagittal aberration, respectively. The “vector” slit at the detector plane converges to a spot whenever the aberrations are corrected. For example, when $\Delta =0^\circ$, the zeroth-order reflection beam from the grating has minimal aberrations. However, the first-order diffracted beam has aberrations proportional to the diffraction angle (Fig. 1c). On the other hand, we show, for the first time, that a toroidal mirror reflection at a misaligned azimuthal angle tuned to $\Delta =2^\circ$ can introduce aberrations with a sign and magnitude that cancel the distortions caused by the grating, as shown in Fig. 1d–f for wavelengths close to 30 nm. Note that the aberrations of the zeroth-order beam exhibit slight distortions at this azimuthal angle (Fig. 1f).

Hence, the tangential and sagittal aberrations due to the toroidal mirror and the diffraction grating have opposite signs and can cancel each other under specific configurations to achieve a near-perfect focus at the CCD plane for a selected harmonic wavelength. More details can be found in the Supplementary Materials (SM). Analytically, we derive a simple expression for the aberration-free diffracted beam with a wavelength of interest $\lambda$ as the toroid is tuned to an angle $\Delta$:

2 $$\Delta =\frac{m\lambda }{4d\sin \gamma }$$

For small azimuthal angles, Eq. (2) can be simplified as:

3 $$4\Delta =\frac{m\lambda }{d\sin \gamma }=\sin \alpha +\sin \beta \approx \alpha +\beta$$

Experimentally, the aberration-free spectrometer configuration becomes notably simpler and fully symmetric when both $\alpha =2\Delta$ and $\beta =2\Delta$. Effectively, the compensation of the aberrations can be streamlined to an adjustment of a single azimuthal angle $\Delta =\alpha /2=\beta /2$. The procedure involves two steps: first, determine the location of the zeroth-order reflected beam, and second, tune the azimuthal angle $\Delta$ to shift the first-order harmonic beam to the original position of the zeroth-order. This straightforward approach can be a basis for automated aberration-free monochromators applicable to various of imaging and spectroscopic applications.

### Experimental characterization of the efficiency and resolution of the aberration-free dual-optic spectrometer–monochromator

**Fig. 2:**
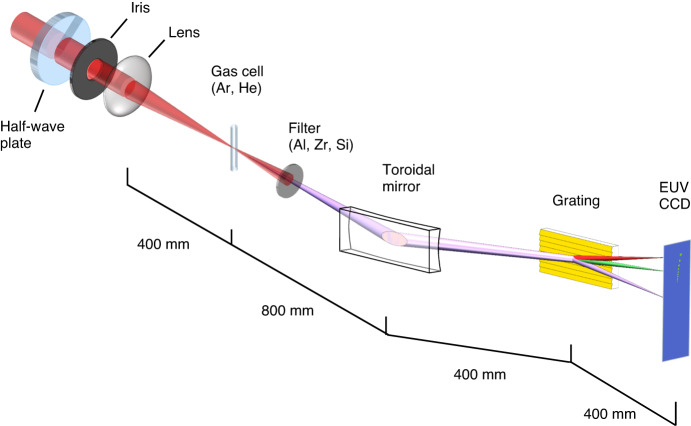
Experimental characterization of the high-performance EUV—soft X-ray spectrometer—monochromator using high harmonic generation light. Intense femtosecond near-infrared laser pulses of 1036 nm or 800 nm are focused into a gas cell filled with argon to generate EUV light or helium for X-ray light. The coherent high harmonic beams are imaged in a 2*f*–2*f* configuration by a toroidal mirror with a focal length of *f* = 400 mm, then spectrally resolved by a grating set at an incident angle *γ* = $6^\circ$. The conical diffraction is detected by an EUV CCD camera with a pixel size of 20 × 20 μm

EUV broadband combs of high order harmonics with wavelengths of 12–45 nm are produced by focusing 1036 nm Yb: KGW or 800 nm Ti:Sapphire laser pulses down to a 100 and 150 μm spot, respectively, in a gas cell filled with a noble gas (see Fig. 2). The co-propagating infrared beam is eliminated using aluminum thin-film filters. Then the broadband harmonic beam is imaged in a 2$f$–2$f$ configuration using a focusing gold-coated toroidal mirror. Subsequently, the EUV beam is angularly dispersed using a plane gold-coated grating in a conical diffraction geometry placed at half the distance between the toroidal mirror and an EUV CCD camera. To evaluate the transmission of the system, a zero-order half-waveplate is used to switch between the S and P polarization states. Table 2 summarizes the optical parameters of the monochromator and spectrometer.

**Table 2:** Parameters of the spectrometer

Parameter	Value
Toroid focal length $f$	400 mm
Altitude (incident) angle *γ*	6°
Tuning (azimuthal) angle Δ	0°–4°
Simulated wavelength range	12–45 nm

**Fig. 3:**
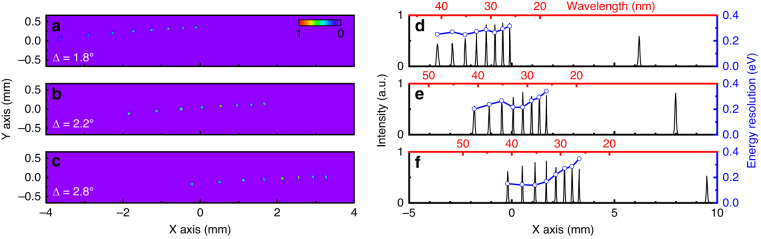
Experimental optimization of spectral resolution and aberration-free spatial profiles of coherent EUV high harmonic beams. **a**–**c** EUV combs of harmonics at 28–41 nm or 30–44 eV. The azimuthal angle Δ of the toroidal mirror is rotated to focus different harmonic beams at the center of the detector (*X* = 0) where spatial aberrations are eliminated. The graphs show perfectly round spatial profiles of the optimized **a** 39th (26.6 nm), **b** 33rd (31.4 nm) and **c** 25th (41.4 nm) harmonics near the center of the CCD for $\Delta =$ 1.8°, 2.2°, 2.8° respectively. **d**–**f** Normalized EUV spectral intensity showing averaged efficiency greater than 40% and an excellent energy resolution of $\lambda /\Delta \lambda$ > 200 at these angles. For example, the resolution at 25th harmonics of 41.4 nm in (**f**) is 0.15 eV, or $\lambda /\Delta \lambda$ > 200 at 30 eV

First, we characterize the performance of the system in the EUV region using seven high-order harmonics ranging from the 25th to 39th, covering the wavelength range of 28–41 nm. To optimize the spectral resolution, the beam spot of each harmonic order is minimized at the detector plane. As we adjust the azimuthal angle $\Delta$ of the toroid from 0° to 1.8°, 2.2° and 2.8°, we refocus the 39th, 33rd, and 25th harmonics consecutively in the first diffraction order near the center of the detector, where their aberrations are eliminated, as demonstrated in Fig. 3a–c. All seven harmonics exhibit excellent spectral resolution of sub-0.2 eV. Specifically, in this instrument configuration, the linewidth of the 25th harmonics at 41 nm is $\lambda /\Delta \lambda$ > 200, corresponding to 0.15 eV resolution at 30 eV. In this context, the design can readily serve as a monochromator by positioning a vertical slit at *X* = 0 and adjusting the angle $\Delta$ to isolate a specific wavelength with minimal aberration. We note that altering this angle results in a minor Y-directional displacement of the output spot, as evidenced by the 39th harmonic focusing at *Y* > 0 in Fig. 3a and the 25th harmonic at *Y* < 0 in Fig. 3c. This spatial shift can be effectively counterbalanced by slightly adjusting the grating’s position along the X-axis, thereby recentering the harmonic beam spot at *Y* = 0 without inducing significant aberrations. A secondary toroidal or ellipsoidal mirror can then be employed to refocus and demagnify the isolated monochromatic beam spot, reducing its size for more precise applications.

Our ray tracing simulations estimate that this high-resolution spectrometer can focus a point source to a perfect spot size of ~20 μm, or the limit of one-pixel size of the CCD, for the experimentally measured harmonic divergence of 1.2 mrad. This is the theoretical spatial extent of the point spread function of the optical system. Experimentally, the FWHM beam diameter of the 39th harmonic at 26 nm (Fig. 3a) is only ~3 pixels, corresponding to ~60 μm. Factors like surface and slope imperfections of the grating and toroid can contribute to the enlarged beam size observed in the image plane.

One of the most remarkable advantages of using a grating in conical diffraction geometry is its high diffraction efficiency. For a grating with blazing angle $\psi$, the diffraction efficiency reaches the maximum when the incident light and the diffracted light specularly reflect off the groove surface. Then the angles satisfy the following relationship:

4 $$\alpha +\beta =2\psi$$

In analogy to the conventional grating orientation, the diffraction efficiency of the conical blazed grating will also be reduced by a shadowing effect—if the incidence plane is not perpendicular to the groove surface, each groove diffracts only a portion of the harmonic light because the partially shadowed area by the adjacent grooves. The shadowing effect is minimized for $\alpha =\psi$. Therefore, according to Eq. (3), the highest possible efficiency is achieved at a wavelength satisfying $\alpha =\beta =\psi =2\Delta$, which is the Littrow condition combined with the angle for aberration corrections. It is worth noting that the shadowing effect can be minimized if the blaze angle $\psi$ is selected to be significantly small.

**Fig. 4:**
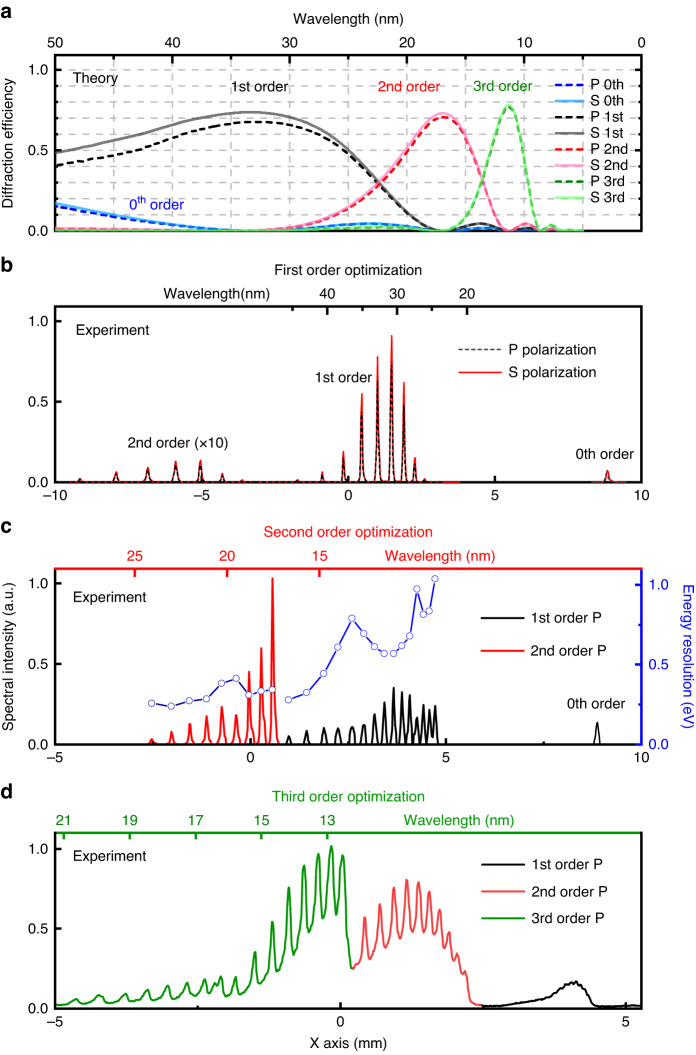
Extremely high grating efficiency in all first, second, and third diffraction orders and high spectral resolution at EUV and soft X-ray wavelengths. **a** Theoretical absolute grating efficiency of 68–78% from coupled-mode simulations in a conical geometry. The solid and dashed lines represent the high efficiency of the S and P polarizations, respectively, preserving the angular momentum state of the light, especially at shorter wavelengths or higher diffraction orders. **b** Highest experimental efficiency of P-polarized high harmonics covering 26–41 nm in the first diffraction order. **c** Highest experimental efficiency of P-polarized high harmonics covering 17–33 nm spectral range in the second (red line) order and its corresponding energy resolution of sub-0.34 eV or $\lambda /\Delta \lambda$ ~ 200 at the optimized wavelengths at the center of the CCD. **d** Highest experimental efficiency of P-polarized high harmonics covering 12-20 nm spectral range in the third diffraction order (green line)

Figure 4a shows the calculated grating’s diffractive efficiency for the P and S polarization states of the first four diffraction orders in the spectral range of 10–50 nm, using a coupled-mode theory^32–35^. We assume ideal gold-coated surfaces with no contamination or surface roughness. The highest efficiencies are obtained at three wavelengths: 32.6 nm, 16.97 nm, and 11.3 nm. For the first-order diffraction, the maximum efficiency is 67.7% for P polarization and 73.6% for S polarization at 32.6 nm. For the second-order diffraction, the maximum efficiency is 70.6% for P polarization and 72.9% for S polarization at 16.97 nm. For the third-order diffraction, the maximum efficiency is 77% for P polarization and 77.9% for S polarization at 11.3 nm. Essentially, the first, second, and third diffractive order can be used to cover the entire spectral range from 10 to 50 nm at near-constant high efficiency using a single grating.

The theoretically calculated diffractive efficiencies are in excellent agreement with the experimental measurements in the EUV region. The experiments are first conducted at the 26–41 nm spectral range. The relative signal strength between the zeroth, first, and second-order is plotted in Fig. 4b. For spectral signals near 30 nm, the maximum diffraction efficiency falls into the first order, which agrees well with the theoretical calculations. Next, the HHG signal is measured without a grating at the 26 to 41 nm spectral range to acquire the absolute diffraction efficiency. We measured the absolute grating efficiency to be 50.4% in the first order. In excellent agreement with the experimental data, the theoretically averaged diffraction efficiency of the gold-coated grating for harmonics in the 26–41 nm spectral range is 61.5% for the P and 66.4% for the S polarization. In principle, it is possible to further increase the absolute diffraction efficiency by using a smaller grazing angle geometry and selecting an appropriate blaze angle without modifying the geometry of the spectrometer.

The efficiencies of the HHG beam with P and S polarization are measured by changing the driving laser polarization with a half-wave plate. The experimental and theoretical data suggest that the difference between the S and P polarization signal strength is less than 10% in the EUV and significantly decreases to near zero towards the soft X-ray region or for higher diffractive orders. (Fig. 4a and Fig S1 (SM)). Thus, this spectrometer scheme is ideal for preserving the beam polarization or angular momentum of the HHG beam.

The broad tunability of the spectrometer is affirmed by employing high-order harmonic spectra within the shorter EUV wavelength range, spanning from 17 to 33 nm. These harmonics are generated in helium gas. We achieve maximum efficiency at the second-order diffraction at this wavelength, while the 0th and 1st orders are suppressed (Fig. 4c), in line with our theoretical predictions (Fig. 4a red). An increased tuning-aberration-correction angle of $\Delta$ = 3° is used to optimize the harmonic beams near 17 nm, allowing us to attain an impressive energy resolution of 0.34 eV at 17.6 nm (71 eV), equivalent to a relative resolution of $\lambda /\Delta \lambda$ = 209. This results in an ideal beam quality for high-resolution imaging applications, when configured as a monochromator.

To extend the efficiency measurements toward soft X-ray wavelengths and test the superior performance at the 3rd diffraction order, harmonics in the 12–20 nm spectral range are produced using Ti:Sapphire driving pulses focused on helium gas. The infrared light is filtered with zirconium and silicon thin-film foils. For coherent emission near the silicon ${L}_{2}$ and ${L}_{3}$ absorption edges at ~100 eV, the 3rd order is confirmed to have the highest diffraction efficiency (see Fig. 4a, d), while the 1st and 2nd diffraction orders are weaker. The reduced spectral resolution near 13 nm is caused by spectrum overlapping between 2nd- and 3rd-order signals, which can be corrected computationally.

Practically, this demonstrates that the first, second, and third orders, etc., from a single grating can be implemented in various spectrometer-monochromator designs. Higher orders are expected to have similar performance. In a global optimization, the designs can cover a broader soft X-ray spectrum by selecting the most efficient diffraction order, and by optimizing the corresponding azimuthal angle of a toroid.

### Temporal characteristics of the dual-optic spectrometer–monochromator

**Fig. 5:**
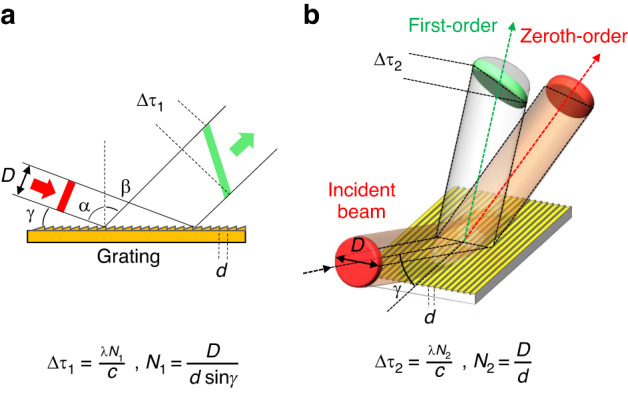
Temporal broadening of the diffracted EUV beam caused by pulse front tilt. **a** Conventional diffraction geometry. **b** Conical diffraction geometry. Here, $\Delta {\tau }_{i}$ is pulse broadening caused by pulse front tilt measured as a tail-to-tail spread of the intensity envelope of the pulse, *D* is the diameter of the incident beam, *d* is the grating groove spacing, *N*_*i*_ is the total number of grooves illuminated within the beam diameter, $\gamma$ is the beam grazing angle, *c* is the speed of light and $\lambda$ is the wavelength of interest

An essential application of high-order harmonic sources is studying ultrafast dynamics in materials, including nanodevices, magnetic materials, and advanced quantum nano-systems. In such applications, maintaining the short high order harmonic pulse duration is important when transmitting the HHG beam through multiple optical elements, i.e., focusing and diffractive optics, multilayer mirrors. Diffraction gratings introduce a significant amount of spatial and temporal distortions that can drastically increase the pulse duration. Pulse broadening caused by the chromatic dispersion of complex optical systems can be analyzed using a perturbative approach—the Lah–Laguerre optical formalism (LLOF)^36^. In the case of a single grating, the broadening $\Delta \tau$ is mainly due to the diffracted pulse front tilt^37^, as shown in Fig. 5a.

In general, the broadening is proportional to the total number of grooves ${N}_{1}$ illuminated within the beam diameter $D$, and increases with the decrease of the grazing angle $\gamma$. In the case of the conical diffraction shown in Fig. 5b, for small $\gamma$, the total number of illuminated grooves ${N}_{2}$ is invariant of grazing angle $\gamma$, resulting in less pulse broadening when compared to the conventional grating geometry^31^. Further reducing the pulse broadening down to <10 fs requires using grating pairs^38–42^.

**Fig. 6:**
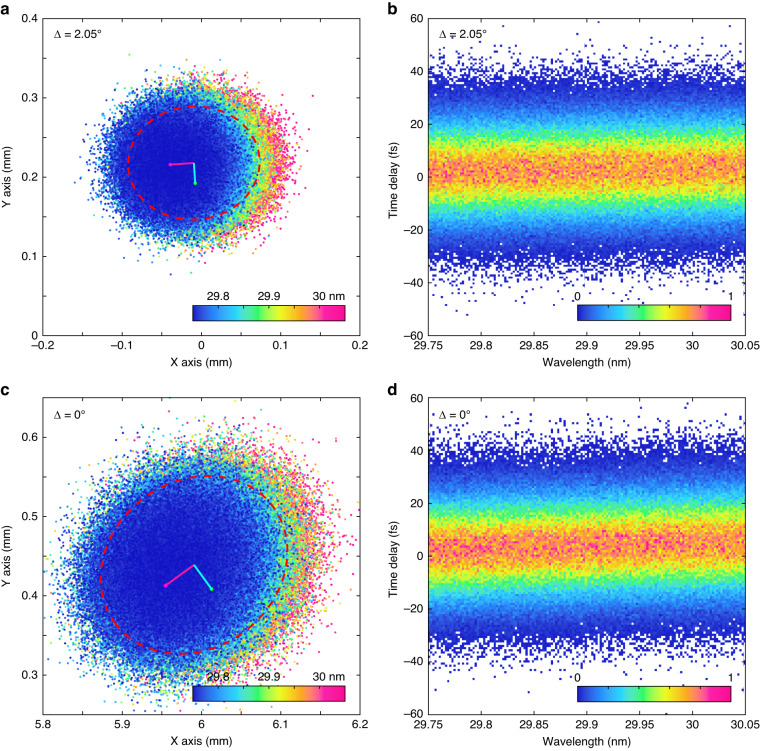
Temporal spread of the diffracted beam in the first diffraction order of the globally optimized monochromator when the grating is placed at a distance from the toroid. Statistical Monte-Carlo ray tracing of an isolated harmonic at 29.9 ± 0.15 nm with color-coded wavelength distribution at the exit of the monochromator with aberration corrections at an azimuthal toroidal angle of **a** $\Delta =$ 2.05° versus the unoptimized geometry **c** $\Delta =$ 0°. The dashed elliptical line encloses the $2\sigma$ confidence area. **b** Extracted temporal pulse broadening from Monte-Carlo ray tracing for $\Delta =$ 2.05°. The broadening is mainly caused by pulse front tilt having a tail-to-tail spanning of 80 fs with a standard deviation of ${\sigma }_{\tau }$ = 11.9 fs, corresponding to FWHM duration of 29 fs. **d** Temporal pulse broadening from Monte-Carlo ray tracing for $\Delta =$ 0°, showing the larger slope and curvature of the time delay as a function of the wavelength. The false color in b and d correlates with the intensity of the histogram counts

To quantitatively assess the pulse spatio-temporal characteristics of our dual-optic spectrometer-monochromator, we employed Monte-Carlo ray tracing. This approach allows us to precisely characterize the spatial and temporal EUV beam parameters in a computationally efficient manner, utilizing a modest number of rays. Our simulation utilized a fully coherent statistical source at 29.9 nm (41.4 eV), with a flat top spectrum of ±0.15 nm, 30 μm standard deviation spatial width extent (with beam spot ellipsoid parameters $a=$ 70.06 μm, $b=$ 69.55 μm, measured at $2\sigma$, enclosing ~95.5% of the data points), and a statistical divergence of ${\sigma }_{\theta }=$ 0.6 mrad standard deviation up to $3{\sigma }_{\theta }$. In addition, we optimized the system’s geometry with a tilted toroid at 2.05° to minimize temporal aberrations at this specific wavelength. In the temporal domain, a plane wave beam is broadened by the spectrometer to a 80 fs tail-to-tail pulse width with ${\sigma }_{\tau }=$ 11.9 fs standard deviation, corresponding to an FWHM of 29 fs as Fig. 6b shows. The ray tracing beam spot diagram in Fig. 6a displays the distortion-corrected, spatial beam spot distribution at focus. The ellipsoid parameters, measured at $2\sigma$, are $a=$ 81.6 μm and $b=$ 70.63 μm at focus, representing 1:1 imaging despite the presence of the aberrations from the dispersive element and the tilted toroid. Further, as a comparison, a light source with the same beam parameters but with a divergence of ${\sigma }_{\theta }=$ 0.1 mrad standard deviation (a representative number for waveguide-generated HHG) results in pulse spread with ${\sigma }_{\tau }=$ 2 fs standard deviation and less than 11 fs tail-to-tail spread. The spectrometer/monochromator under these conditions has a theoretical wavelength resolution of 5 nm/mm. The spot diagram in Fig. 6c illustrates the noncorrected spatial profile distribution at focus with ellipsoid parameters of $a=$ 122.7 μm while $b=$ 104.5 μm (measured at $2\sigma$).

**Fig. 7:**
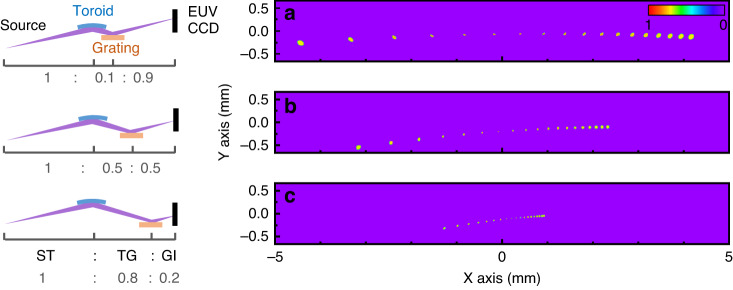
Theoretical simulations illustrating the spectral resolution of the spectrometer and the EUV—X-ray pulse broadening through adjusting the position of the grating. **a** The spectral resolution and the temporal pulse broadening can be optimized by adjusting the distance GI between the grating and the image on the CCD. A larger GI gives a higher resolution at the cost of a significant pulse broadening (**a** TG < GI). **b**, **c** Conversely, a smaller GI offers a lower resolution but also a smaller pulse broadening over a broader spectral range (**b** TG = GI, **c** TG > GI). To correct for aberrations, the angle $\Delta$ in **a**–**c** equals 3.6°, 2.0°, and 0.8°, respectively, and for all configurations, the aberration-free focused beams are near the center of the CCD

Since the amount of pulse broadening is proportional to the laser beam diameter on the grating, in our spectrometer design, one can vary the distance between the focusing mirror and the grating to adjust the beam diameter on the grating. In Fig. 7, we investigate the impact of the distortions introduced by the grating at various distances. The CCD records the combined aberrations that result from the toroidal reflection and the grating diffraction. Here, we first decompose the two contributions. The toroidal distortions are proportional to the tuning angle $\Delta$ and the propagation distance from the toroid to the image on the CCD, (see Eq. (S3) in SM). In contrast, the distortions from the conical diffraction are proportional to the sum of the angles of diffraction $\alpha +\beta$ and the propagation distance from the grating to the CCD (see Eq. (S1) in SM). Interestingly, as a result, the optimized beam is located at the center of the CCD even if the grating is not centered between the toroid and the CCD (see Fig. 7). Compensating the aberrations leads to:

5 $$\frac{\alpha }{\beta }=\frac{\rm{GI}}{\rm{TG}}$$

6 $$\frac{\alpha +\beta }{4\Delta }=\frac{\rm{ST}}{2\rm{GI}}$$

Since $\alpha =2\Delta ,$ we then have:

7 $$\beta =2\Delta \frac{\rm{TG}}{\rm{GI}}$$

where ST, TG, and GI are the distances from the source to the toroid, from the toroid to the grating, and from the grating to the imaging plane of the CCD, respectively. For completeness, in the symmetric configuration shown in Fig. 7b, the grating is positioned in the middle TG = GI or ST = 2TG = 2GI, which leads to $\alpha +\beta =4\Delta$, consistent with Eq. (3).

Considering the finite size and pixel density of commercial EUV CCDs, the spectrometer mode of operation can function at the highest spectral resolution by increasing the grating to CCD distance GI (Fig. 7a) at the cost of a significant temporal pulse broadening. Alternatively, by decreasing the distance GI (Fig. 7c), we can reduce the temporal pulse broadening and increase the spectral range that fits on the CCD chip. This mode of operation can better serve as a monochromator for ultrafast femtosecond pump–probe experiments.

## Discussion

In summary, we have developed a flat-field, highly efficient conical-diffraction imaging spectrometer with exceptional performance characteristics, without employing variable line spacing grating. By using a single toroidal mirror and a single grating, we measure a throughput efficiency of more than 40% over a broad bandwidth of harmonics. Furthermore, the theoretical throughput efficiency for the entire system free of imperfections, such as surface roughness and contamination, surpasses 60% in all orders. The high throughput efficiency is also accompanied by a superior spectral resolution of *λ*/Δ*λ* > 200 over a broad EUV—X-ray spectral range covered using a single grating and its efficient low and high diffraction orders.

One of the most compelling key advantages of the design is the capability to compensate for spatial aberrations of the selected harmonic beam caused by the conical diffraction grating with a simple azimuthal rotation of the toroidal focusing mirror relative to the grating. This approach ensures optimal imaging performance over a broad spectral range while maintaining circular and elliptical polarization states at the first and especially at the second and third diffraction orders.

Furthermore, our analyses indicate small temporal dispersion of the pulse broadening with a tail-to-tail intensity confined to 80 fs with a FWHM value of 29 fs and a standard deviation of $\sigma =$ 11.9 fs. This level of performance enables ultrafast spectroscopic and pump–probe experiments with femtosecond-to-picosecond precision, providing researchers with a powerful tool for studying a wide range of micro-scale dynamics.

Finally, the improved understanding of the physical optical phenomena related to imaging distortions has resulted in designing an instrument with optimized spatio-temporal parameters, facilitating advanced and versatile EUV—X-ray measurements. This robust and practical design is ideal for use in both tabletop coherent EUV—X-ray light sources as well as next-generation attosecond sources at large-scale facilities^43^. Our work opens up exciting new avenues for a wide range of scientific investigations, from fundamental studies of light–matter interactions and dynamics in atoms, molecules, plasmas, solids, and strong electron–electron correlations, to applied research in areas such as plasma diagnostics, astrophysics, materials science, chemistry, and biology.

## Materials and methods

### Laser systems and coherent high-order harmonic generation

The bright phase-matched high-harmonic combs in the extreme ultraviolet range are generated in noble gases using two different near-infrared laser amplifiers. The signal from 17 to 41 nm shown in Fig. 3, Fig. 4b, c, is obtained using a 1036 nm, 200 fs Yb: KGW 6 W laser system (PHAROS, Light Conversion) with pulse energy up to 1.5 mJ. The laser beam is focused to a diameter of 100 μm using a 400 mm focal length lens. We use argon gas and aluminum filters to phase-match the signal in the range of 25–41 nm shown in Fig. 3 and Fig. 4b, or helium gas to generate a harmonic signal extending to a higher phase-matching cutoff in the range of 17 nm to 25 nm, as shown in Fig. 4c. We use aluminum thin film filters to remove the IR light. The shorter-wavelength high harmonic signal from 12 nm to 20 nm shown in Fig. 4d is generated using an 800 nm, 30 fs Ti:Sapphire laser amplifier with pulse energy of up to 3 mJ. The laser beam is focused to a diameter of 150 μm, using a 400 mm focal length lens, into a gas cell filled with helium gas to generate phase phase-matched high-harmonics at 12–20 nm. We use zirconium thin film filters to remove the IR light.

### Simulation of dual-optic spectrometer-monochromator

The focusing optical element in the spectrometer–monochromator is a gold-coated toroidal mirror with a focal length of 40 cm. The toroid has a high reflectivity of 77–83% and a relatively large numerical aperture at $\gamma$ = 6° glancing incidence. The gold-coated conical-diffraction grating, which is also set at $\gamma$ = 6°. The grating diffracts the incident beam into a circularly symmetric ring pattern (conical diffraction) with high angular dispersion and a measured efficiency greater than 50.4%.

Theoretically, the grating efficiency at the various diffraction orders is evaluated using the coupled mode wave analysis method. This framework effectively solves Maxwell’s equations for periodic structures by representing the incident field and the grating as Fourier series. The diffraction efficiencies are the power ratios of each diffracted order to the incident power.

The ray tracing simulations are performed using Zemax and Matlab software. We have developed a Monte-Carlo ray tracing to optimize the spatial and temporal features of the optical system. This method quickly and accurately simulates the beam propagation with a small number of rays. We use statistically distributed random parameters for the starting beam profile and treat the reflectivity as a random statistical problem while the rays are handled in the geometric optics approximation.

### Supplementary information

Supplemental material

## References

[1] McPherson A (1987). Studies of multiphoton production of vacuum-ultraviolet radiation in the rare gases. J. Opt. Soc. Am. B.

[2] Li XF (1989). Multiple-harmonic generation in rare gases at high laser intensity. Phys. Rev. A.

[3] Krause JL, Schafer KJ, Kulander KC (1992). High-order harmonic generation from atoms and ions in the high intensity regime. Phys. Rev. Lett..

[4] Corkum PB (1993). Plasma perspective on strong field multiphoton ionization. Phys. Rev. Lett..

[5] Rundquist A (1998). Phase-matched generation of coherent soft X-rays. Science.

[6] Chang ZH (1997). Generation of coherent soft X rays at 2.7 nm using high harmonics. Phys. Rev. Lett..

[7] Popmintchev T (2012). Bright coherent ultrahigh harmonics in the keV x-ray regime from mid-infrared femtosecond lasers. Science.

[8] Popmintchev D (2015). Ultraviolet surprise: efficient soft X-ray high-harmonic generation in multiply ionized plasmas. Science.

[9] Shan B, Chang Z (2001). Dramatic extension of the high-order harmonic cutoff by using a long-wavelength driving field. Phys. Rev. A.

[10] Gaumnitz T (2017). Streaking of 43-attosecond soft-X-ray pulses generated by a passively CEP-stable mid-infrared driver. Opt. Express.

[11] Li J (2017). 53-attosecond X-ray pulses reach the carbon K-edge. Nat. Commun..

[12] Kraus PM (2018). The ultrafast X-ray spectroscopic revolution in chemical dynamics. Nat. Rev. Chem..

[13] Žutić I, Fabian J, Das Sarma S (2004). Spintronics: fundamentals and applications. Rev. Mod. Phys..

[14] Miao JW (2015). Beyond crystallography: diffractive imaging using coherent X-ray light sources. Science.

[15] Zhang BS (2015). High contrast 3D imaging of surfaces near the wavelength limit using tabletop EUV ptychography. Ultramicroscopy.

[16] Popmintchev D (2018). Near- and extended-edge X-ray-absorption fine-structure spectroscopy using ultrafast coherent high-order harmonic supercontinua. Phys. Rev. Lett..

[17] Klemke N (2019). Polarization-state-resolved high-harmonic spectroscopy of solids. Nat. Commun..

[18] Kfir O (2015). Generation of bright phase-matched circularly-polarized extreme ultraviolet high harmonics. Nat. Photonics.

[19] Klas R (2021). Ultra-short-pulse high-average-power megahertz-repetition-rate coherent extreme-ultraviolet light source. PhotoniX.

[20] Li J (2020). Attosecond science based on high harmonic generation from gases and solids. Nat. Commun..

[21] Cavalieri AL (2007). Attosecond spectroscopy in condensed matter. Nature.

[22] Wang H (2010). Attosecond time-resolved autoionization of argon. Phys. Rev. Lett..

[23] Nakano N (1984). Development of a flat-field grazing-incidence XUV spectrometer and its application in picosecond XUV spectroscopy. Appl. Opt..

[24] Koike M, Yamazaki T, Harada Y (1999). Design of holographic gratings recorded with aspheric wave-front recording optics for soft X-ray flat-field spectrographs. J. Electron Spectrosc. Relat. Phenom..

[25] Hettrick MC, Underwood JH (1986). Stigmatic high throughput monochromator for soft X rays. Appl. Opt..

[26] Poletto L (2004). Instrumentation for analysis and utilization of extreme-ultraviolet and soft X-ray high-order harmonics. Rev. Sci. Instrum..

[27] Nicolosi P (2005). Grazing-incidence spectrometer for the monitoring of the VUV FEL beam at DESY. J. Electron Spectrosc. Relat. Phenom..

[28] Cash W (1982). Echelle spectrographs at grazing incidence. Appl. Opt..

[29] Pascolini M (2006). Gratings in a conical diffraction mounting for an extreme-ultraviolet time-delay-compensated monochromator. Appl. Opt..

[30] Sie EJ (2019). Time-resolved XUV ARPES with tunable 24–33 eV laser pulses at 30 meV resolution. Nat. Commun..

[31] Frassetto F (2011). Single-grating monochromator for extreme-ultraviolet ultrashort pulses. Opt. Express.

[32] Derrick GH (1979). Crossed gratings: a theory and its applications. Appl. Phys..

[33] Greffet JJ, Baylard C, Versaevel P (1992). Diffraction of electromagnetic waves by crossed gratings: a series solution. Opt. Lett..

[34] Bräuer R, Bryngdahl O (1993). Electromagnetic diffraction analysis of two-dimensional gratings. Opt. Commun..

[35] Johnson KC (2014). Projection operator method for biperiodic diffraction gratings with anisotropic/bianisotropic generalizations. J. Opt. Soc. Am. A.

[36] Popmintchev D (2022). Analytical Lah-Laguerre optical formalism for perturbative chromatic dispersion. Opt. Express.

[37] Poletto, L., Villoresi, P. & Frassetto, F. Diffraction gratings for the selection of ultrashort pulses in the extreme-ultraviolet. in (ed Grishin, M.) *Advances in Solid State Lasers: Development and Applications* (Vukovar, Croatia: IntechOpen, 2010), 413–438.

[38] Frassetto F, Villoresi P, Poletto L (2008). Optical concept of a compressor for XUV pulses in the attosecond domain. Opt. Express.

[39] Eckstein, M. et al. Alignment and characterization of the two-stage time delay compensating XUV monochromator. Preprint at *arXiv* https://arxiv.org/abs/1604.02650 (2016).

[40] Poletto L (2009). Time-delay compensated monochromator for the spectral selection of extreme-ultraviolet high-order laser harmonics. Rev. Sci. Instrum..

[41] Poletto L (2007). Intense femtosecond extreme ultraviolet pulses by using a time-delay-compensated monochromator. Opt. Lett..

[42] Frassetto, F. & Poletto, L. Design of a compact time-delay-compensated monochromator for femtosecond pulses in the extreme-ultraviolet. Proc SPIE 12581, X-Ray Free-Electron Lasers: Advances in Source Development and Instrumentation VI. (SPIE, Prague, Czech Republic, 2023).

[43] Kühn S (2017). The ELI-ALPS facility: the next generation of attosecond sources. J. Phys. B.

[44] Ito M (2010). Spatiotemporal characterization of single-order high harmonic pulses from time-compensated toroidal-grating monochromator. Opt. Express.

[45] Igarashi H (2012). Pulse compression of phase-matched high harmonic pulses from a time-delay compensated monochromator. Opt. Express.

[46] Ito K (2019). Polarimetry of a single-order circularly polarized high harmonic separated by a time-delay compensated monochromator. Opt. Express.

