# Supplementary material for: Highly efficient and aberration-free off-plane grating spectrometer and monochromator for EUV—soft X-ray applications
Source: Light Sci Appl. 2024 Jan 8;13:12. doi: 10.1038/s41377-023-01342-9 (PMC10772113; doi:10.1038/s41377-023-01342-9)
Supplement: Supplementary file 1 — Supplemental material [file 41377_2023_1342_MOESM1_ESM.docx]

Supplementary Materials for

****Highly efficient and aberration-free off-plane grating spectrometer and monochromator for EUV**** – ****soft X-ray applications****

Jie Li^1,2^, Kui Li^1,2^, Xiaoshi Zhang^1,2,3^, Dimitar Popmintchev^4^, Hao Xu^1^, Yutong Wang^1^, Ruixuan Li^1,2^, Guangyin Zhang^1,2^, Jiyue Tang^1,2^, Jin Niu^1,2^, Yongjun Ma^1^, Runyu Meng^2,5^, Changjun Ke^1,2^, Jisi Qiu^1,2^, Yunfeng Ma^1,2^, Tenio Popmintchev^4,6^ and Zhongwei Fan^2^

^1^Aerospace Information Research Institute, Chinese Academy of Sciences, Beijing 100094, China

^2^School of Optoelectronics, University of the Chinese Academy of Sciences, Beijing 100049, China

^3^Yunnan University, Kunming, Yunnan 650500, China

^4^Photonics Institute, TU Wien, Vienna A-1040, Austria

^5^Yunnan Observatories, Chinese Academy of Sciences, Kunming, Yunnan 650011, China

^6^University of California, Physics Department, San Diego, La Jolla, CA 92093, USA

Corresponding authors: ^1^[lijie430@aircas.ac.cn](mailto:lijie430@aircas.ac.cn), ^3^zhangxiaoshi@itc.ynu.edu.cn, ^4^[dimitar.popmintchev@gmail.com](mailto:dimitar.popmintchev@gmail.com), ^6^[tenio.popmintchev@physics.ucsd.edu](mailto:tenio.popmintchev@physics.ucsd.edu), ^2^fanzhongwei@ucas.ac.cn

These authors contributed equally: Jie Li, Kui Li

**This file includes:**

Supplementary Text

Eqs. S1 to S7

Fig. S1

In these Supplementary Materials, we provide additional data to support the core findings presented in the main manuscript. We first provide a quantitative evaluation of the diffraction aberrations arising from the grating in a conical diffraction geometry. Subsequently, we derive the aberrations induced by including a toroidal mirror. Finally, we evaluate the throughput efficiency of the system using Monte Carlo simulations, focusing specifically on an optical assembly that combines a toroidal mirror with a grating in a conical diffraction configuration.

Supplementary Note 1: Analytical Relations for the Grating-Induced Aberrations in Conical Diffraction Geometry at Grazing Incidence Angle

A toroidal mirror introduces a gradually converging beam, which leads to more pronounced aberrations in the grating diffraction in contrast to a collimated beam (see Fig. 1a). In this Supplementary Note 1, we will provide a comprehensive quantitative evaluation of these diffractive aberrations. As we will demonstrate in Supplementary Note 2, these aberrations can be effectively mitigated through strategic tuning of the toroidal mirror.

To investigate the aberration from conical diffraction, we set the toroid at its default angle ($\Delta=0^{\circ}$). The object and image distance are both equal to twice the focal length$ST=TG+GI=2f$, where $ST$, $TG$, $GI$ are the distances from the source to the toroid, from the toroid to the grating, and from the grating to imaging plane of the CCD, respectively. Without loss of generality, we assume $TG=GI=f$.

A ray tracing is performed to analyze the behavior of the ${23}^{\mathrm{th}}$ to ${59}^{\mathrm{th}}$ harmonics from a $1036 nm$ driving laser field. A total of $19$ odd harmonic peaks are observed, separated by $2.39 eV$. In terms of wavelength, the spectrum covers the $17-45 nm$ range. Our simulation uses a $10 \mu m$ pixel size of the detector to record the beam spot size.

In the case of zeroth-order diffraction ($m=0$), rays emitted at an angle of $\alpha=0$ and diverging at an angle of $2\theta$ uniformly converge at the central point of the detector. For the first-order diffraction ($m=1$), according to Eq. (1), different harmonics are diffracted at varying azimuthal angles $\beta$, as illustrated in Fig. 1b. It is evident that the dispersed harmonics fail to converge to a singular focal point, in contrast to the zeroth-order scenario. This beam spread, as depicted in Fig. 1c, can be attributed to the diffractive behavior governed by Eq. (1).

We investigate the spatial spread at each wavelength by simplifying the source divergence along the sagittal (horizontal direction along X) and tangential (vertical direction along Y) planes. To achieve this, vector slits are positioned along the X and Y directions in front of the toroid, creating distinct sagittal and tangential ray fans (see Fig. 1a). The slit length A equals $4\theta f$. The slits are marked with red and yellow arrows to indicate the direction. The beam spot of the sagittal and tangential rays on the detector is also marked with corresponding arrows to show the aberration direction of each ray fan in Fig. 1c. The sagittal ray fan (the horizontal slit, the red arrow in Fig. 1a) at the source point is diffracted into a vertical line on the detector. The tangential ray fan (the vertical slit, the yellow arrow) is diffracted into a horizontal line at a small tilt angle β, which matches the direction of the semicircle described by the azimuthal angle in the conical diffraction geometry.

We further examine the illuminated area on the grating surface. For sagittal rays, the azimuthal angle ranges from $-\theta$ to $+\theta$ and the intersection position on the grating surface varies from $-\theta f$ to $+\theta f$ along the $X$ direction. By applying an analytical ray tracing, we find the length of the vertical image in Fig. 1c is equal to the vertical projection of the conical diffraction of the sagittal slit (width of $2\theta f$) at the azimuthal angle $\beta$. The length of the vertical image can be calculated as:

$$\begin{aligned} L_{\mathrm{grating}}=2\theta f\left( \sin\alpha+\sin\beta\right) \#\left( S1 \right) \end{aligned}$$

The aberration caused by the grating is proportional to the angle of deviation due to the diffraction ($\sin\alpha+\sin\beta$) and the propagation distance after the grating ($f$). Assuming $\alpha=0$ and $\gamma\approx\sin\gamma$, we obtain:

$$\begin{aligned} 2\theta f\left( \sin\alpha+\sin\beta\right)=\frac{2\theta f\lambda}{d\gamma}\#\left( S2 \right) \end{aligned}$$

Analyses of the length scale of the image of the tangential rays, gives the same result of $2\theta f\lambda/d\gamma$. Here the image of the tangential rays has a small tilt angle $\beta$*.* Therefore, the simplified equation for determining the beam spot diameter across varying harmonic orders can be expressed as approximately $2\theta f\lambda/d\gamma$. Finally, if the high harmonic beam contains a certain amount of angular distribution, in this configuration, one can record the beam divergence profile $2\theta(\lambda)$ for each individual harmonic.

Supplementary Note 2: Aberrations from the Toroidal Mirror at Grazing Incidence Angle

The toroidal mirror introduces specific aberrations that can be analyzed analytically. In this study, we demonstrate that the aberrations arising from a toroidal reflection can counterbalance the aberrations from a grating.

To analyze the toroidal aberrations, we orient the toroid at a deviation angle of $\Delta$ and calculate the location of the zeroth-order reflection, emanating from the grating. The result is shown in Fig. 1d and f. As earlier, we utilize two vector slits to distinguish the diffusion direction of the tangential and sagittal ray fans. With a non-zero tilt *Δ*=2°, the zeroth-order reflected beam，as captured by the detector, exhibits a lateral shift on the sensor array, as illustrated in Fig. 1d. We also find that the sagittal ray fan (the horizontal slit, the red arrow in Fig. 1a.) emitted from the point source converges to a vertical line on the detector.

On the other hand, the tangential ray fan (the vertical slit, the yellow arrow) converges to a horizontal line with a slight tilt. Surprisingly, the direction of the arrows is opposite to that of the diffraction grating. Using analytical ray tracing, the diameter of the spot (or the length of the lines) introduced by the toroidal tuning angle $\Delta$ is determined to be:

$$\begin{aligned} L_{\mathrm{toroidal}}=8\Delta f\theta\#\left( S3 \right) \end{aligned}$$

Since the aberrations caused by the diffraction grating and the toroid have opposite signs, the toroidal angle $\Delta$ and the grating parameters can be tuned to minimize the spatial beam diffusion for a wavelength of interest. The total aberration reaches a minimum if the following condition is satisfied:

$$\begin{aligned} 8\Delta f\theta=\frac{2\theta f\lambda}{d\gamma}\#\left( S4 \right) \end{aligned}$$

which can be simplified to:

$$\begin{aligned} 4\Delta=\frac{\lambda}{d\gamma}=\sin\alpha+\sin\beta\approx\alpha+\beta\#\left( S5 \right) \end{aligned}$$

where we have assumed that $\alpha$, $\beta$, and $\gamma$ are small angles.

*Equation (S5) illustrates the conditions required to achieve both minimal beam spot size and highest resolution.* By varying the toroidal angle $\Delta$, the reflected beam after the toroid will reach the grating at an azimuthal angle $\alpha=2\Delta$. The azimuthal angle of the diffracted beam, determined by Eq. (1), can be reduced to a more straightforward expression as follows:

$$\begin{aligned} \beta\left( \lambda\right)=\frac{\lambda}{d\gamma}-2\Delta\#\left( S6 \right) \end{aligned}$$

In other words, one can select a specific angle $\Delta$ so that the wavelength of interest, denoted as $\lambda$, fulfills Eq. (S5). Given that $\alpha=2\Delta,$ we obtain that the aberration-corrected targeted wavelength is diffracted to an azimuthal angle $\beta(\lambda)=2\Delta$ (Eq. (S6). In this arrangement, we have a perfectly symmetric geometry for the conical diffraction: $\alpha=\beta=2\Delta$, where the diffracted beam is located at the center of the detector. Therefore, tuning the angle $\Delta$ ensures the aberration-eliminated harmonic beam will always be diffracted towards the center of the detector, as illustrated in Fig. 1d and e. Such mode of operation is ideal for monochromators.

Finally, a raytracing simulation is performed to verify our analyses (Fig. 1d). By setting the $\Delta=2^{\circ}$, the ${35}^{th}$ harmonic ($30 nm$) is diffracted (in the first order) to the center of the detector with a minimum spot size of ~$25 \mu m$ (Fig. 1e), which is now limited by higher order aberrations. Such spatial resolution matches approximately the minimal pixel spacing of 15-25 μm of the standard HHG detectors, such as EUV – X-ray CCD cameras or microchannel plates with similar channel spacing.

Using a first-order approximation, we can calculate the spot diameter of the adjacent harmonics near the ${35}^{\mathrm{th}}$:

$$\begin{aligned} D\left( \lambda\right)=4f\theta\left( \beta\left( \lambda\right)-\beta\left( \lambda_{0} \right) \right)\#\left( S7 \right) \end{aligned}$$

where $\beta(\lambda_{0})=2\Delta$ is the azimuthal angle of the optimized harmonic, and $\beta\left( \lambda\right)$ is the azimuthal angle of the adjacent harmonics. The beam spot increases linearly with the angle deviation from $\beta\left( \lambda_{0} \right)$, as in the case of $\Delta=0^{\circ}$ in Fig. 1b.

Supplementary Note 3: Efficiency Throughput of the Optical Design of the Spectrometer and Monochromator

We developed Monte Carlo ray tracing analyses to evaluate the throughput efficiency of the integrated optical system, comprising a toroidal mirror and a grating set in a conical diffraction geometry.

In our simulations, we utilize a fully coherent statistical source characterized by variable photon energy and a flat-top spectral distribution within each harmonic beam, with an energy bandwidth ratio of $E/\Delta E=200$. We consider an extended source featuring a spatial beam width with a standard deviation of $30 \mu m$ (beam spot ellipsoid parameters $a =70.06 \mu m$, $b =69.55 \mu m$, measured at the $2\sigma$ level that encompasses approximately 95.5% of the data points) and a statistical divergence having a standard deviation of $\sigma_{\theta}=0.6 mrad$, extending up to $3\sigma$.


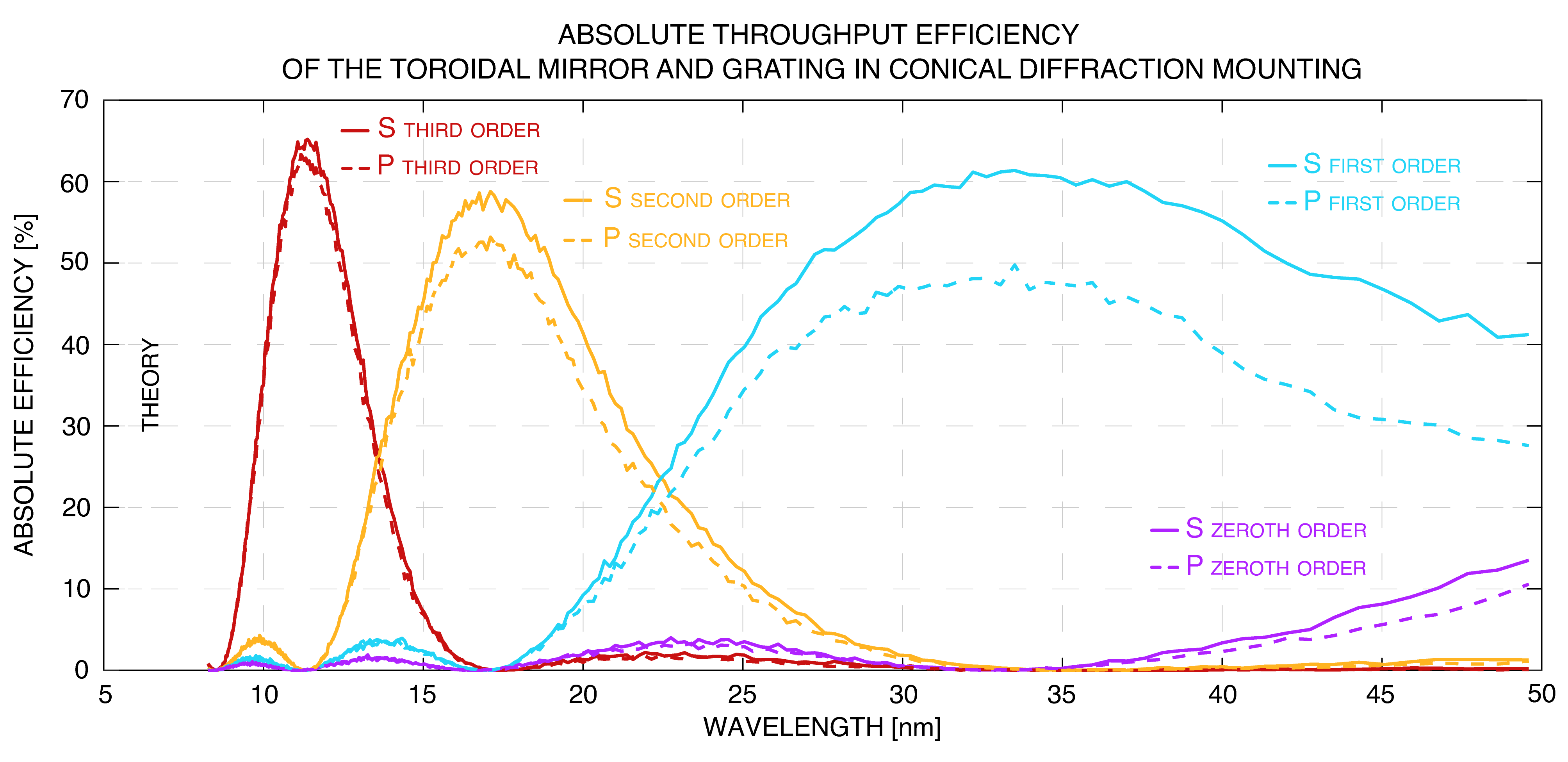


**Fig. S1 Spectrometer efficiency in the zeroth, first, second, and third-order diffractions, illustrating identical efficiencies for S and P polarizations at higher order diffractions.** Absolute transmission of the S and P polarizations in different diffraction orders through the optical system, calculated using a Monte Carlo ray tracing for an energy interval of $E/\Delta E = 200$. The solid and dashed lines represent the S and P polarizations for the first four diffraction orders in matching colors. The simulation accounts statistically for the reflectivity of the toroidal mirror and the diffraction efficiency of the grating.

Throughout the simulations, we assume ideal gold-coated surfaces without contamination or manufacturing defects, such as surface roughness, slope errors, etc. The results in Fig. S1 demonstrate an outstanding maximum efficiency exceeding 60%.

The plotted data results from 2000 Monte Carlo ray tracing simulations of the optical design. The grating efficiency metrics are obtained from coupled-mode Fourier analyses and then treated statistically in a ray tracing stochastic model. For the first-order diffraction, the maximum throughput efficiency is 49.8% for P polarization and 61.3% for S polarization at 33 nm. For the second-order diffraction, the maximum throughput efficiency is 53.2% for P polarization and 58.7% for S polarization at 17 nm. Finally, for the third-order diffraction, the maximum throughput efficiency is 63.3% for P polarization and 65.2% for S polarization at 11.3 nm.

One more notable advantage of this spectrometer-monochromator configuration is its reduced zeroth-order intensity relative to the higher-order diffraction intensities, allowing for simultaneous observation of all orders on an X-ray CCD camera. In contrast, the detector is usually saturated by the presence of an intense zeroth-order beam in a typical setting.
